# Polypeptide N-acetylgalactosaminyltransferase 2 regulates cellular metastasis-associated behavior in gastric cancer

**DOI:** 10.3892/ijmm.2012.1130

**Published:** 2012-09-18

**Authors:** DONG HUA, LI SHEN, LAN XU, ZHI JIANG, YINGHUI ZHOU, AIHUAN YUE, SHITAO ZOU, ZHIHONG CHENG, SHILIANG WU

**Affiliations:** 1 The Fourth Affiliated Hospital of Soochow University, Wuxi, Jiangsu 214062;; 2 Department of Biochemistry and Mollecular Biology, School of Medicine, Soochow University, Suzhou, Jiangsu 215123;; 3 Department of Biochemistry and Molecular Biology, Hubei University of Medicine, Shiyan, Hubei 442000, P.R. China

**Keywords:** polypeptide N-acetylgalactosaminyltransferase 2, gastric cancer, metastasis, matrix metalloproteinase-2, transforming growth factor-β1

## Abstract

Aberrant glycosylation of cell surface glycoprotein due to specific alterations of glycosyltransferase activity is usually associated with invasion and metastasis of cancer, particularly of gastric carcinomas. Polypeptide N-acetylgalactosaminyltransferase 2 (ppGalNAc-T2), which catalyzes initiation of mucin-type O-glycosylation, is also involved in tumor migration and invasion. However, a comprehensive understanding of how ppGalNAc-T2 correlates with the metastasic potential of human gastric cancer is not currently available. In the present study, ppGalNAc-T2 was detected in a variety of human poorly differentiated tumor cells, and expression appeared to be higher in SGC7901 gastric cancer cells. In addition, we investigated the potential effects of ppGalNAc-T2 on growth and metastasis-associated behavior in SGC7901 cells after stable transfection with ppGalNAc-T2 sense and antisense vectors. We found that cell proliferation, adhesion and invasion were decreased in ppGalNAc-T2 overexpressed cells but increased in ppGalNAc-T2 downregulated cells. Therefore, we attempted to clarify the mechanisms underlying the anti-metastatic activities of ppGalNAc-T2. Further investigation indicated that overexpression of ppGalNAc-T2 is involved in the inhibition of matrix metalloproteinase (MMP)-2 expression at both the protein and mRNA levels, which may be associated with ppGalNAc-T2 suppressing the expression of transforming growth factor (TGF)-β1. However, it did not exhibit any apparent correlation with MMP-14 expression levels. Our data show the effect of ppGalNAc-T2 on proliferation, adhesion or invasion of SGC7901 gastric cancer cells, suggesting that ppGalNAc-T2 may exert anti-proliferative and anti-metastatic activity through the decrease of MMP-2 and TGF-β1. These results indicate that ppGalNAc-T2 may be used as a novel therapeutic target for human gastric cancer treatment.

## Introduction

Cancer cells frequently exhibit alterations in protein glycosylation when compared to their normal counterparts and this is assumed to result from disruptions in expression levels or activity of the enzymes of glycosylation, the glycosyltransferases and glycosidases (1). Aberrant glycosylation of membrane components due to specific alterations of glycosyltransferase activity is a common feature of carcinoma cells and is usually associated with invasion and metastasis of cancer. For example, GCNT2, which is a gene-encoding glucosaminyl (N-acetyl) transferase 2, contributes to breast cancer metastasis with preferential expression in basal-like breast cancer (2). ST6Gal-I expression in ovarian cancer cells promotes an invasive phenotype by altering integrin glycosylation and function (3). Since aberrant glycoproteins as a result of these enzymes may be involved in promoting tumor invasion and metastasis, these enzymes could also be used as cancer biomarkers (4). Therefore, cancer-specific changes in the expression of glycosyltransferases exhibit the most marked and consistent change of activity in tumorigenesis.

Gastric cancer is the fourth most common malignancy and the second leading cause of cancer-related mortality in the world. Several reports indicate a complexity in glycosyltransferase activities which lead to several tumor associated carbohydrate structures in gastric carcinoma. Glycosyltransferase mRNA expression has been found to be significantly altered in gastric carcinomas isolated from surgical specimens (5). Upregulation of glycan:sulfotransferase activities and downregulation of α,2-fucosyltransferase activity appear to be associated with human gastric tumori-genesis (6). Shimizu *et al*(7) confirmed that α4GnT, which forms a unique glycan, GlcNAcα1-->4Galβ-->R, is detectable in 80% of 5 patients with an early stage of gastric cancer and the expression level of α4GnT mRNA is increased in association with tumor progression. Recently, β3Gn-T8, which can extend polylactosamine on N-glycan, was also reported to be involved in malignancy in gastric cancer cells (8). However, our previous study demonstrated that polypeptide N-acetylgal actosaminyltransferase 2 (ppGalNAc-T2) was also involved in gastric cancer migration and invasion.

ppGalNAc-T2 is a member of the ppGalNAc-T family which catalyzes the attachment of the first N-acetylgalactosamine (GalNAc) monosaccharide to the polypeptide at the initiation of O-linked glycosylation of proteins. All ppGalNAc-Ts in mammals are type II transmembrane proteins that have a Golgi lumenal region that contains a catalytic domain with glycosyltransferase activity and a C-terminal R-type lectin domain (9). The human ppGalNAc-T family contains more than 18 members, each of which has unique transferase activity, different peptide substrate specificities, and dissimilar patterns of expression (10,11). Among all the ppGalNAc-Ts identified in mammals thus far, the ppGalNAc-T2 gene was highly expressed in cancer and may play an important role in the occurrence and development of tumor. Brooks *et al*(1) reported that levels of ppGalNAc-T2 expression may change with the differentiation of breast carcinoma. Mandel *et al*(12) showed that ppGalNAc-T2 expression was strong in poorly differentiated tumors. It has also been confirmed that ppGalNac-T2 is involved in vanadium-induced HL-60 cell differentiation (13). Moreover, both acute T cell leukemia Jurkat cell lines and human heptocarcinoma HepG-2 cell lines clearly express ppGalNAc-T2 (14). The invasion and metastasis of human glioma cells can also be regulated by ppGalNAc-T2 (15). Our previous studies revealed that ppGalNAc-T2 expression appears to be higher in gastric cancer SGC7901 cells than in other poorly differentiated human cancer cells, indicating that ppGalNAc-T2 may play a vital role in the process of gastric cancer emergence and development. However, a comprehensive understanding of how ppGalNAc-T2 correlates with the invasive potential of human gastric cancer is not currently available.

Numerous studies have shown that overexpression of Matrix metalloproteinases (MMPs) is correlated with the progression of gastric cancer, which contributes to tumor invasion, metastasis and angiogenesis. Thus, this study was undertaken to evaluate the role of ppGalNAc-T2 in gastric cancer invasion and metastasis by creating stable transfectants and evaluating them for invasive and metastatic potential *in vitro*. In order to elucidate the role of ppGalNAc-T2 in the gastric cancer metastasis process, SGC7901 cells were treated with ppGalNAc-T2 sense or antisense vectors and examined for the following: i) the relationship between the ppGalNAc-T2 expression and the cell proliferation, adhesion, and invasion ability, in order to clarify whether ppGalNAc-T2 is correlated with SGC7901 cell metastasis-associated behavior; ii) the impact of ppGalNAc-T2 on MMP-2, MMP-14 and transforming growth factor (TGF)-β1 regulation including mRNA and protein levels, to investigate the molecular mechanisms of the anti-metastasic activities in human gastric carcinoma. These data suggested that high expression of the ppGalNAc-T2 gene in gastric cancer SGC7901 cells might exert anti-growth and anti-metastasic activity through the decrease of MMP-2 and TGF-β1. Our findings indicate that ppGalNAc-T2 is useful in regulating gastric carcinoma invasion and metastasis, and this may be used as a novel approach for cancer therapy.

## Materials and methods

### Cell culture

The SGC7901 human gastric cancer, SHG44 glioma, SHI-1 leukemia, A549 lung adenocarcinoma, and HO8910 ovarian cancer cell lines were obtained from Shanghai Cell Bank (Shanghai, China). They were cultured in RPMI-1640 (Gibco, USA) containing 10% fetal bovine serum (FBS) in a humidified atmosphere with 5% CO_2_ at 37°C. They were selected as all these poorly differentiated cells often have aberrant terminal sugar structures of O-glycan chains.

### Generation and selection of cells stably transfected with pEGFP-C1-ppGalNAc-T2 sense vectors and pEGFP-C1-ppGalNAc-T2 antisense vectors

Transfection was carried out using Lipofectamine™ 2000 (Invitrogen, Carlsbad, USA), according to the manufacturer’s instructions. SGC7901 cells (2x10^5^) were plated onto 6-well plates until they reached 70–90% confluency before transfection. Cells were transfected with 4 μg of pEGFP-C1-ppGalNAc-T2 sense vectors (SGC7901-T2s group) and pEGFP-C1-ppGalNAc-T2 antisense vectors (SGC7901-T2as group), followed by selection with G418 (500 μg/ml). Individual clones were isolated and expanded for further characterization. The empty vector pEGFP-C1 was also transfected into SGC7901 cells and served as the control group. Transfection efficiency was detected by fluorescence microscopy (Zeiss; Gottingen, Germany). All plasmids were constructed and conserved in our laboratory (13,15).

### Cell proliferation assay

Cell proliferation was measured with MTT assay. Briefly, SGC7901 cells and the stably transfected clones were plated in 96-well plates at a density of cells (5x10^3^) and 180 μl culture medium was added to each well. The cells were incubated at 37°C for 24, 48, 72, 96 or 120 h, at which time the cells were incubated with 100 μl of MTT solution (5 g/l; Sigma, St. Louis, MO, USA) for 4 h. The reaction was stopped by the addition of l50 μl DMSO (Sigma) and the absorbance of samples at 570 nm was then measured. A growth curve was plotted for each sample as the log cell number vs. time, and the growth rates were derived from the slope of each growth curve. Three independent experiments were performed and the results were used for plotting the relative growth rate with SD.

### In vitro cell adhesion assay

The adhesion of SGC7901 cells stably transfected with sense or antisense ppGalNAc-T2 vectors was performed using standard methods. A flat-bottomed 96-well plate was coated overnight at 4°C with 0.2 ml Matrigel (200 μg/ml). Some wells were left uncoated as negative control. The plate was washed twice with phosphate-buffered saline (PBS), blocked with 1 mg/ml bovine serum albumin (BSA) for 2 h at 37°C and then 0.5 ml suspension of tumor cells (5x10^3^) were added. After the plate was incubated at 0.5, 1 and 1.5 h intervals at 37°C, unattached cells were removed by washing with PBS. MTT was added to each well and the absorbance value obtained by seeding uncoated wells represented 100% adhesion and all other values were divided by this to calculate percentage adhesion.

Furthermore, to investigate the adhesion of cells to various extra-cellular matrix (ECM) components, 96-well plates were precoated with either 1 mg/ml hyaluronic acid (HA) or 50 1 g/ml fibronectin (FN). The adhesive ability of gastric cancer cells was also detected as described above.

### In vitro cell invasion assay

The invasiveness of different SGC7901 stable cells was evaluated in 24-well Transwell chambers (Costar Corporation, Cambridge, MA, USA), according to the manufacturer’s instructions. Briefly, Transwell chambers equipped with polycarbonate membrane (12 mm pore size) were precoated with 6.25 mg/l Matrigel on the upper chamber. The cells were cultured in serum-free medium for 12–24 h. Cells (1x10^5^) were seeded in each transwell insert containing 200 μl of serum-free medium with BSA. Then 500 μl of culture medium with 10% FBS was added into each well of a 24-well plate. The cells and Matrigel on the upper chamber were removed using a cotton stick after 12 h. Cell penetration through the membrane was quantified by counting the number of cells that penetrated the membrane in ten microscopic fields (at x200 magnification) per filter. The experiment was repeated 3 times.

### RNA isolation and reverse transcription-polymerase chain reaction (RT-PCR)

Total-RNA was isolated from equal cell numbers using Tri Reagent (Sigma) following the manufacturer’s instructions. Reverse transcription was as previously described and 5 μl of the resultant cDNA was used as template for PCR (16). The sequences for primers with annealing temperatures indicated in brackets were as follows: ppGalNAc-T2, 5′-AAGAAAGACCTTCATCACAGCAATGGAGAA-3′ (forward) and 5′-ATCAAAACCGCCCTTCAAGTCAGCA-3′ (reverse) (60°C); MMP-2, 5′-AGATCTGCAAACAGGACA TTGTATT-3′ (forward) and 5′-TTCTTCTTCACCTCATTG TATCTCC-3′ (reverse) (56°C); MMP-14, 5′-TGGCGGGTGA GGAATAAC-3′ (forward) and 5′-GGGAACGCTGGCAGT AGAG-3′ (reverse) (56°C); TGF-β1, 5′-TGTGGCTACTGGT GCTGAC-3′ (forward) and 5′-ATAGATTTCGTTGTGGG TTTC-3′ (reverse) (56°C); β-actin, 5′-CATGTACGTTGCTA TCCAGGC-3′ (forward) and 5′-CTCCTTAATGTCACGCA CGAT-3′ (reverse) (52°C). The number of PCR cycles used was 30 and the expected product size after primer amplification was as follows: ppGalNAc-T2, 669 bp; MMP-2, 332 bp; MMP-14, 690 bp; TGF-β1, 317 bp; and β-actin, 330 bp. The PCR products were separated by electrophoresis on 10 g/l agarose gels and visualized by ethidium bromide staining.

### Western blot analysis

To detect ppGalNAc-T2, MMP-2, MMP-14 and TGF-β1 protein expression in gastric cancer cells after the indicated treatment, cells were harvested and extracted using the standard methods. Equal amounts of protein (50 μg) were separated by SDS-PAGE and transferred to a polyvinylidene difluoride membrane. The membrane was blocked with 5% skim milk in Tris-buffered saline and then incubated with primary antibodies for 1 h at room temperature. The proteins were analyzed using specific antibodies as indicated. Horseradish peroxidase (HRP)-conjugated secondary antibodies and an enhanced chemiluminescence (ECL) kit were used for detection. Anti-human ppGalNAc-T2 monoclonal antibody was produced from rabbits in our laboratory (14). Anti-β-actin rabbit mAb, anti-MMP-2 rabbit mAb, anti-MMP-14 rabbit mAb and anti-TGF-β1 rabbit mAb as well as the anti-rabbit second antibody were purchased from Santa Cruz Biotechnology, Inc. (Santa Cruz, CA, USA).

### Statistical analysis

The results shown are the mean ± SD. A P-value <0.05 was considered to indicate statistically significant differences. Statistical analyses were calculated using SPSS 11.5. Each experiment was repeated 3 times.

## Results

### Expression of ppGalNAC-T2 mRNA in human poorly differentiated malignant tumor cells

**Figure 1:**
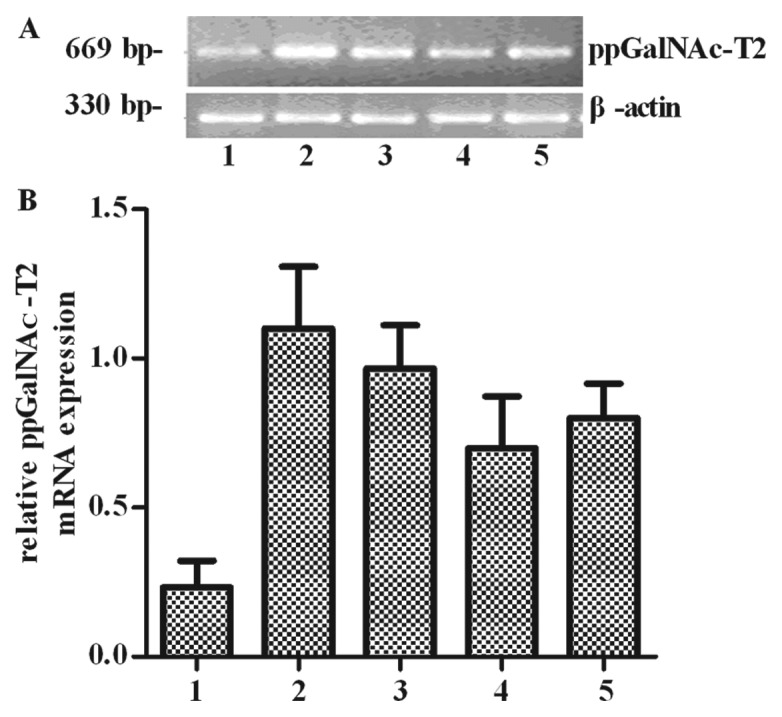
Expression of ppGalNAc-T2 mRNA in human tumor cells. (A) The mRNA level of ppGalNAc-T2 was detected by RT-PCR. (B) The intensity of PCR product was normalized against β-actin. 1, SHG44 cells; 2, SGC7901 cells; 3, SHI-1 cells; 4, A549 cells; and 5, HO8910 cells.

To investigate the potential role of ppGalNAc-T2 in human malignant tumors, we first detected the expression panel of ppGalNAc-T2 in 5 types of poorly differentiated malignant tumor cell lines. The mRNA level of ppGalNAc-T2 in these cells was determined by RT-PCR. All poorly differentiated tumor cells which have aberrant terminal sugar structures of O-glycan chains, including SGC7901 gastric cancer, SHG44 glioma, SHI-1 leukemia, A549 lung adenocarcinoma, and HO8910 ovarian cancer cells expressed ppGalNAc-T2 (Fig. 1). Thus ppGalNAc-T2 may be markers for poorly differentiated carcinomas. In addition, we found that ppGalNAc-T2 mRNA was differentially expressed, as shown in Fig. 1A. The mRNA expression ratios (ppGalNAc-T2/β-actin) were 0.38±0.016, 1.23±0.017, 0.82±0.035, 0.69±0.014 and 0.78±0.023, respectively. The results showed that ppGalNAc-T2 expression in SGC7901 cells was higher than in other cells (P<0.05) (Fig. 1B), suggesting that this gene may play a key role in gastric tumorigenesis. We therefore used gastric cancer as our research model to determine whether ppGalNAc-T2 is correlated with cell invasion and metastasis.

### Establishment of ppGalNAc-T2 overexpression or downregulation of cells

**Figure 2:**
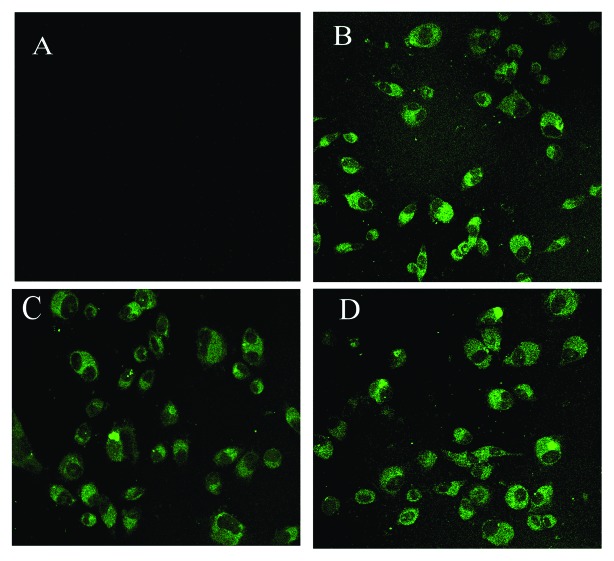
Detection of transfection efficiency using fluorescence microscopy at x200 magnification. (A) Untreated SGC7901 cells; (B) Control group; (C) SGC7901-T2s group; (D) SGC7901-T2as group.

**Figure 3:**
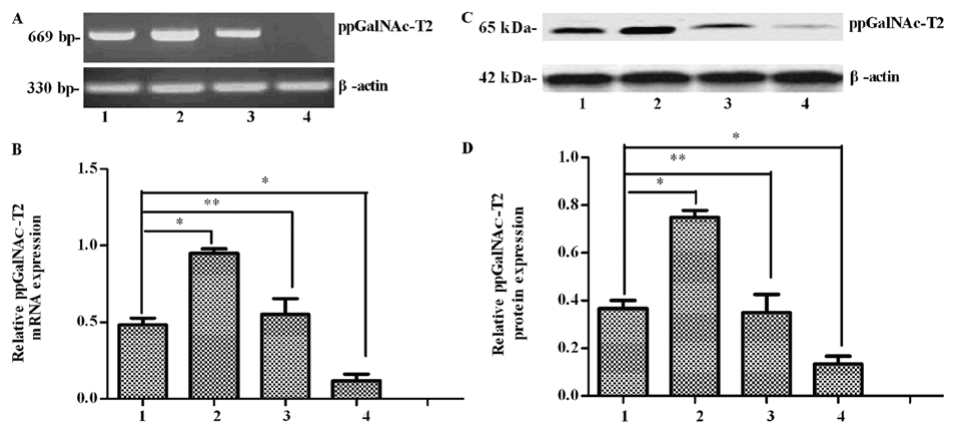
Expression levels of ppGalNAc-T2 in different SGC7901 clones of stably transfected cells, including untransfected SGC7901 cells, SGC7901 cells stably overexpressing ppGalNAc-T2 (SGC7901-T2s), SGC7901 cells with downregulated expression of ppGalNAc-T2 (SGC7901-T2as) or with empty vector as control. β-actin was used as an internal control for loading. ppGalNAc-T2 expression in different SGC7901 cells was analysed by (A) RT-PCR and (C) western blot analysis. (B and D) Band intensity was quantified using densitometry and normalized to β-actin band intensity. 1, Untreated SGC7901 cells; 2, control group; 3, SGC7901-T2s group; and 4, SGC7901-T2as group. (^*^P<0.05, ^**^P>0.05 compared to the untreated group).

To further explore the role of ppGalNAc-T2 in gastric cancer, SGC7901 cells were used to reconstitute the expression of ppGalNAc-T2 by stable overexpression (SGC7901-T2s) or downregulation (SGC7901-T2as) of ppGalNAc-T2. Transfection efficiency was measured using fluorescence microscopy to detect expression of the plasmid-encoded eGFP gene (Fig. 2). Then, the ppGalNAc-T2 mRNA and protein levels in the SGC7901 cells were measured by RT-PCR and western blot analysis, respectively. When compared with untreated cells, ppGalNAc-T2 transcripts were increased in the pEGFP-C1-ppGalNAc-T2 sense vector transfected cells (P<0.05) (Fig. 3A and B). Consistent with the RT-PCR results, the expression of the ppGalNAc-T2 protein was clearly increased in this group (P<0.05) (Fig. 3C and D). Furthermore, ppGalNAc-T2 mRNA and protein expression was suppressed in the SGC7901-T2as group when compared to the untreated group (P<0.05), while no difference was found between the control group and the untreated cells (P>0.05). The above results indicate the successful construction of the ppGalNAc-T2 overexpression or downregulation cell lines. These stable cell lines can be effectively used to further examine the role of ppGalNAc-T2.

### Effect of ppGalNAc-T2 on the viability of SGC7901 cells

**Figure 4:**
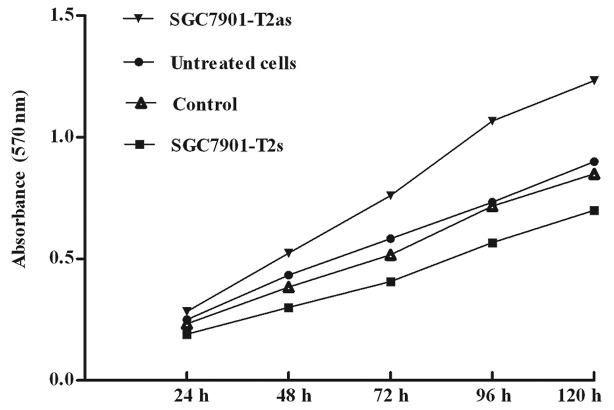
Upregulation of ppGalNAc-T2 inhibits human gastric cancer cell proliferation *in vitro*. SGC7901 cells and their transfectants were cultured in 96-well plates at 5x10^3^/well for 24, 48, 72, 96 and 120 h. Cell growth was assessed by MTT assay.

To examine whether modulation of ppGalNAc-T2 expression affects the tumorigenic properties of the gastric cancer cells, we measured the abilities of *in vitro* cell proliferation by MTT assay. The untreated SGC7901, control, as well as the SGC7901-T2s and SGC7901-T2as cells were grown in culture for 5 days. The ability of cell proliferation in the SGC7901-T2s cells was decreased compared with the control or untreated cells but increased in the SGC7901-T2as cells (P>0.05) (Fig. 4). Treatment of SGC7901 cells with ppGalNAc-T2 sense vectors was associated with a time-dependent inhibition of cell growth, whereas no significant inhibitory effect was observed in the untreated and control cells. These results indicate that multi-step molecular events are necessary for the function of ppGalNAc-T2 to switch the SGC7901 cells from a proliferative state to an inhibited state of cell growth.

### Effect of ppGalNAc-T2 on cell adhesion

**Figure 5:**
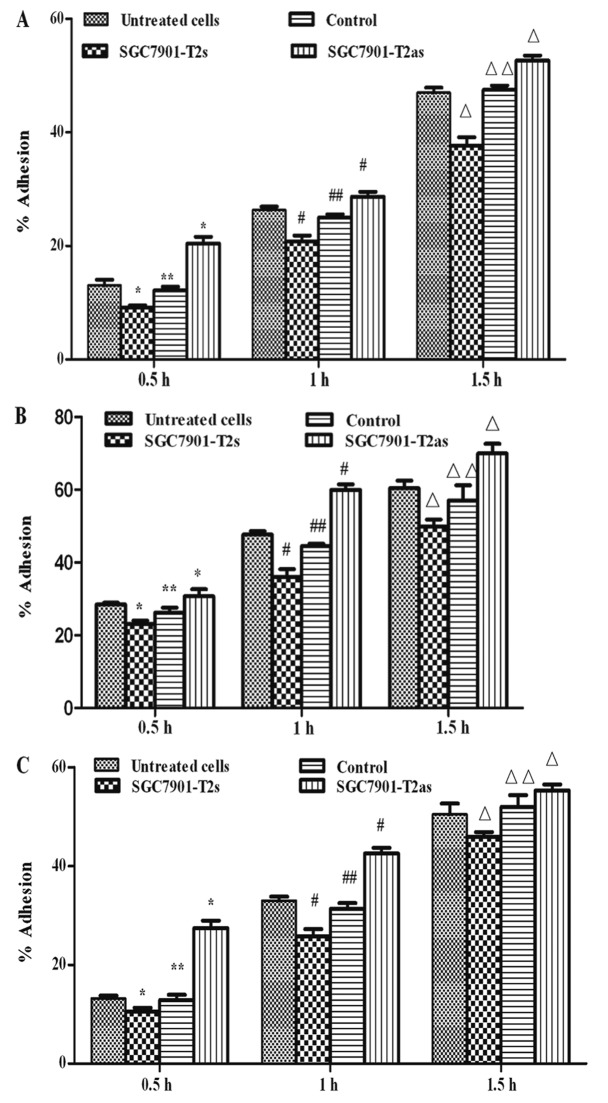
*In vitro* adhesion of SGC7901 cells in the presence of (A) Matrigel, (B) HA and (C) FN at different time points. The cells (5x10^3^) were added to a 96-well plate coated with HA, FN or Matrigel, and the cells were incubated at 0.5, 1 and 1.5 h intervals. The number of attached cells was calculated by the MTT assay. Results showed that overexpression of ppGalNAc-T2 inhibits cell adhesion. Values are expressed as the mean ± SD of three independent experiments. (^*^P<0.05, ^**^P>0.05 compared to the untreated cells at 0.5 h; ^#^P<0.05, ^##^P>0.05 compared to the untreated cells at 1 h; ^▵^P<0.05, ^▵▵^P>0.05 compared to the untreated cells at 1.5 h).

Adhesion is a key event in the metastasic process where cells must first adhere to the ECM prior to its degradation. To examine whether ppGalNAc-T2 expression is associated with adhesion of gastric cancer, *in vitro* adhesion assay was carried out to evaluate the adhesive ability of the untreated SGC7901, control, SGC7901-T2s and SGC7901-T2as cells. The ability of cell adhesion in the SGC7901-T2s group cells was decreased compared with untreated or control SGC7901 cells (P<0.05), but increased in the SGC7901-T2as group cells at different time points (P<0.05) (Figs. 5A, 5B and 5C).

To further investigate the behavior of cells in the presence of ECM components, adhesion assays were carried out in the presence of HA and FN. Increased cell-cell signaling and contact is also mediated by increased expression of cell adhesion molecules. The control, untreated, as well as the SGC7901-T2s and SGC7901-T2as group cells were cultured in the presence of HA and FN. Overexpression of ppGalNAc-T2 led to an average of 32.5% decreased adhesive ability compared with untreated clones at different time points. Conversely, downregulated ppGalNAc-T2 expression caused an average of 58.2% increase in the adhesive ability in the SGC7901-T2as group at different time points (Fig. 5B and C), while no difference was found between the control group and the untreated SGC7901 cells (P>0.05). These results suggest that ppGalNAc-T2 expression is associated with the adhesion of SGC7901 cells *in vitro*; therefore, overexpression of ppGalNAc-T2 has a significant anti-adhesion effect at all intervals.

### Effect of ppGalNAc-T2 on the invasive capability of cells

**Figure 6:**
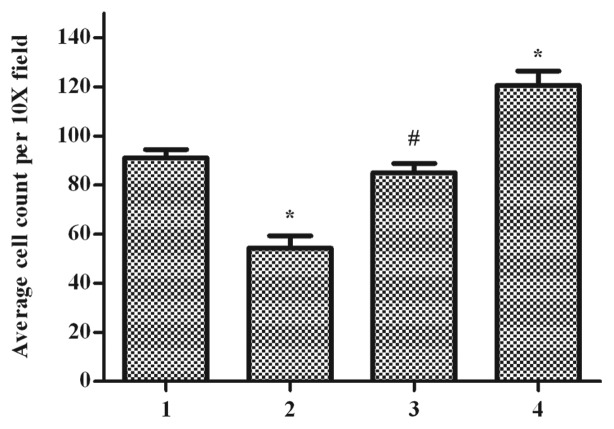
Comparison of *in vitro* invasiveness of cells. The *in vitro* invasion of SGC7901 cells and their transfectants was measured by determined cell counts that penetrated through Matrigel-coated Transwell chambers (12-Am pore size). The experiments are representative of 3 independent experiments with similar results. 1, untreated SGC7901 cells; 2, SGC7901-T2s group; 3, control group; and 4, SGC7901-T2as group. (^*^P<0.05, ^#^P>0.05 compared to the untreated group).

Since ECM degradation is key to tumor cell invasion, the *in vitro* invasiveness of these cell lines through Matrigel coated membranes was compared. Different invasiveness was observed in the control, untreated, as well as the SGC7901-T2s and SGC7901-T2as group cells, respectively (Fig. 6). The control group cells showed little invasion in comparison to the untreated cells (P>0.05), whereas overexpression of ppGalNAc-T2 in SGC7901-T2s cells decreased their migratory capacity (P<0.05). By contrast, downregulation of ppGalNAc-T2 increased the invasive ability in the SGC7901-T2as group (P<0.05). These results suggested that ppGalNAc-T2 expression was inversely associated with the invasiveness of cells *in vitro*. The inverse correlation tendency between ppGalNAc-T2 expression in SGC7901 cells and their *in vitro* invasive ability indicates that ppGalNAc-T2 is likely to be a metastasis suppressor gene in SGC7901.

### Effect of ppGalNAc-T2 on MMP-2 and MMP-14 expression

**Figure 7:**
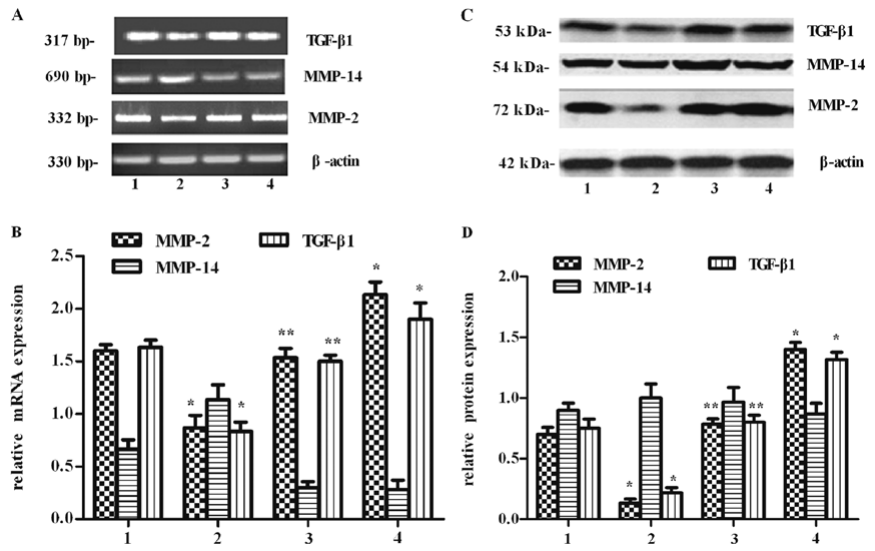
Expression levels of MMP-2, MMP-14 and TGF-β1 in different SGC7901 clones of stably transfected cells, including untransfected SGC7901 cells, SGC7901 cells stably overexpressing ppGalNAc-T2 (SGC7901-T2s), SGC7901 cells with downregulated expression of ppGalNAc-T2 (SGC7901-T2as) or with empty vector as control. β-actin was used as an internal control for loading. ppGalNAc-T2 expression in different SGC7901 cells was analyzed by (A) RT-PCR and (C) western blot analysis. (B and D) Band intensity was quantified using densitometry and normalized to β-actin band intensity. 1, untreated SGC7901 cells; 2, control group; 3, SGC7901-T2s group; and 4, SGC7901-T2as group. (^*^P<0.05, ^**^P>0.05 compared to the untreated group).

Among the MMP family that has been identified, MMP-2 is considered a key enzyme since it is responsible for degradation of the ECM. Meanwhile, MMP-2 activity can be activated by MMP-14, and this activity may be involved in tumor invasion and metastasis. Therefore, to investigate whether the metastasic inhibitory effect of ppGalNAc-T2 resulted from the suppression of MMP-2 and MMP-14 expression, MMP-2 and MMP-14 mRNA and protein levels were measured. Using RT-PCR, we found that the expression of MMP-2 at the mRNA level was lower in the SGC7901-T2s group than in the SGC7901-T2as group (P<0.05), and there was no difference between untreated and control group cells (P>0.05) (Fig. 7A and B). However, there was no evident change on the mRNA transcriptional expression of MMP-14.

The protein levels from whole-cell lysates of MMP-2 and MMP-14 were further assessed by western blot analysis (Fig. 7C and D), respectively. We found that the expression of MMP-2 at the protein level was increased in the ppGalNAc-T2-downregulation cells, but decreased in the ppGalNAc-T2-overexpressing cells (P<0.05). Similar to the RT-PCR results, the expression of the MMP-14 protein presented no noticeable difference in all groups (P>0.05). The changes in the protein levels of MMP-2 and MMP-14 coincided with their mRNA levels, indicating that ppGalNAc-T2 might regulate MMP-2 but not MMP-14 expressions at the transcriptional level.

### Effect of ppGalNAc-T2 on TGF-β1 expression

TGF-β1 promotes tumor progression through the upregulation of MMP-2. To investigate whether the MMP-2 inhibitory effect of ppGalNAc-T2 resulted from the suppression of TGF-β1 expression, TGF-β1 mRNA and protein levels were measured. The SGC7901 cells transfected with ppGalNAc-T2 sense vectors exhibited a direct reduction at the levels of TGF-β1 mRNA and protein (P<0.05) (Fig. 7). Meanwhile, the expression of TGF-β1 in the SGC7901-T2as group was contrary to that in the SGC7901-T2s group (P<0.05). The change of TGF-β1 at the mRNA and protein level displayed a similar trend with that of MMP-2. These results suggest that the MMP-2 inhibitory effect by high expression of ppGalNAc-T2 is probably through regulation of TGF-β1 expression.

## Discussion

Although multiple factors contribute to aberrant glycosylation in cancer, such as the availability and localization of nucleotide sugar donors and substrates, one of the primary mechanisms seems to be the differential expression of glycosyltransferases involved in the synthesis of glycans. Mucin-type linkages (GalNAcα1-O-Ser/Thr) of proteins begin with the addition of a single GalNAc monosaccharide to a serine or threonine residue on the polypeptide. Attachment is catalyzed by a family of glycosyltransferases called the UDP-N-acetylgalactosamine: polypeptide N-acetylgalactosaminyltransferases (ppGalNAc-Ts, EC 2.4.1.41), which is a crucial regulatory step (17,18). These structural changes can alter the function of the cell, and its antigenic and adhesive properties, as well as its potential to invade and metastasize. PpGalNAc-T2, an important member of ppGalNAc-Ts, is often variable in cells of different types and differentiation, and the expression may change in cancer cells (12). Herein, we investigated the mRNA expression of ppGalNAc-T2 in several human poorly differentiated cancer cells, including SGC7901 gastric cancer cells (19), SHG44 glioma cells (20), SHI-1 leukemia cells (21), A549 lung adenocarcinoma cells (22), and HO8910 ovarian cancer cells (23). We found that all of these cells could express ppGalNAc-T2, however, mRNA expression in SGC7901 cells appeared to be higher than in other cells. Furthermore, ppGalNAc-T2 was identified as a metastasis-associated gene, which was highly expressed in human acute T cells, heptocarcinoma HepG-2 cells, and glioma U251 cells (14,15). However, the expression of ppGalNAc-T2 in gastric cancer has not been previously reported. In this regard, we first demonstrated that SGC7901 cells show significantly increased expression of ppGalNAc-T2 compared with other poorly differentiated tumor, suggesting that expression levels of ppGalNAc-T2 in gastric cancer are associated with a high risk of metastasis.

Tumor metastasis occurs by a series of steps including cell attachment, invasion, and proliferation, and is regulated by extremely complicated mechanisms (24,25). For example, patients with gastric cancer generally have metastasis when clinically examined. There is a low 5-year survival rate and poor quality of life even after tumor resection. The SGC-7901 human gastric cell line was first established from the metastatic lymph node of a 56-year-old female patient suffering from gastric adenocarcinoma (26). To examine the potential anti-metastasic effects of ppGalNAc-T2, proliferation, adhesion and invasion assays were performed on SGC7901 cells. Regardless of the exact mechanism of ppGalNAc-T2 in the cell metastatic process, pEGFP-C1-ppGalNAc-T2 sense vectors or pEGFP-C1-ppGalNAc-T2 antisense vectors were transfected into SGC7901 cells to reconstitute the expression of ppGalNAc-T2, focusing on the changes in the characteristics of cell metastasis-associated behavior. In ppGalNAc-T2 overexpressed cells (SGC7901-T2s group), proliferation, adhesion, and invasion were decreased compared with untreated SGC7901 cells, whereas the values for the same assays were increased in ppGalNAc-T2 downregulated cells (SGC7901-T2as group).

Since adhesion is considered a key in regulating cell growth at the metastatic secondary site (27,28), in this study we first proved ppGalNAc-T2 expression affected the adhesive ability of SGC7901 cells by *in vitro* cell adhesion assay. In addition, the degradation of basal membrane and ECM of primary tumor are crucial steps for tumor invasion and metastasis. We also looked at adhesion of the cells on various ECM components including FN and HA. The ppGalNAc-T2 antisense vectors transfected cells attached better to FN and HA than any other cell line. Thus, upregulated expression of ppGalNAc-T2 is likely to inhibit the growth of the cancer cells in the metastatic sites due to a decrease in cell adhesive ability.

Proteins of the MMP family are involved in the breakdown of ECM in normal physiological processes, such as embryonic development, reproduction, and tissue remodeling, as well as in disease processes, such as arthritis and metastasis (29). Among the many MMPs that have been identified, MMP-2 encodes an enzyme which degrades type IV collagen, the major structural component of basement membranes. MMP-14 activates MMP-2 protein, and this activity may be involved in tumor invasion (30). Thus, we examined the effect of ppGalNAc-T2 on the expression of MMP-2 and MMP-14 in SGC7901 cells. Our study revealed that expression of ppGalNAc-T2 had an inverse correlation with the expression of MMP-2 at the mRNA and protein levels. It is highly likely that ppGalNAc-T2 regulates the proliferation, adhesion, and invasiveness, all of which are essential steps for the establishment of metastasis of SGC7901 cells, due to the regulation of the MMP-2 signaling pathway. However, it did not exhibit any apparent correlation with MMP-14 expression. MMP-14 has been shown to interact with tissue inhibitor of metalloproteinase-2 (TIMP-2) (31). Further experiments are required to determine if ppGalNAc-T2 functions in SGC7901 cells, in part, by TIMP-2 signaling pathways.

The multifunctional cytokine transforming growth factor-β1 (TGF-β1) plays a dual role in the process of carcinogenesis by promoting tumor progression by enhancing migration, invasion and survival of tumor cells. TGF-β1 has been proven to play a key role in activating MMP-2 (32). We also investigated the effect of ppGalNAc-T2 on the expression of TGF-β1 in SGC7901 cells. Consistent with this concept, we confirmed that in the SGC7901-T2s group, TGF-β1 was decreased compared with control or untreated cells, whereas downregulation of ppGalNAc-T2 mRNA and protein levels induced activation of TGF-β1. These results suggest that the MMP-2 inhibitory effect by high expression of ppGalNAc-T2 is probably through regulation of TGF-β1 expression.

In conclusion, the present study demonstrated that high expression of ppGalNAc-T2 significantly inhibits the metastasic ability of SGC7901 human gastric cancer cells. The proposed anti-growth and anti-metastasic mechanisms might be mediated through the inhibition of MMP-2 and TGF-β1. These results indicate that ppGalNAc-T2 is also a metastasis regulation gene of human gastric cancer. We suggest that ppGalNAc-T2 can be used as a novel therapeutic target for human gastric treatment. However, to provide a potential valuable therapeutic strategy for gastric cancer metastasis, it is necessary to further investigate the underlying molecular mechanism of ppGalNAc-T2 in suppressing SGC7901 cell metastasis.
